# Whole cell affinity for 4‐amino‐5‐hydroxymethyl‐2‐methylpyrimidine (HMP) in the marine bacterium *Candidatus* Pelagibacter st. HTCC7211 explains marine dissolved HMP concentrations

**DOI:** 10.1111/1758-2229.70023

**Published:** 2024-10-04

**Authors:** Elizabeth Brennan, Stephen Noell, Edward W. Davis, Stephen J. Giovannoni, Christopher P. Suffridge

**Affiliations:** ^1^ Department of Microbiology Oregon State University Corvallis Oregon USA; ^2^ Thermophile Research Unit, Te Aka Mātuatua | School of Science, Te Whare Wānanga o Waikato University of Waikato Hamilton New Zealand; ^3^ Center for Quantitative Life Sciences Oregon State University Corvallis Oregon USA

## Abstract

Vitamin B1 is a universally required coenzyme in carbon metabolism. However, most marine microorganisms lack the complete biosynthetic pathway for this compound and must acquire thiamin, or precursor molecules, from the dissolved pool. The most common version of Vitamin B1 auxotrophy is for thiamin's pyrimidine precursor moiety, 4‐amino‐5‐hydroxymethyl‐2‐methylpyrimidine (HMP). Frequent HMP auxotrophy in plankton and vanishingly low dissolved concentrations (approximately 0.1–50 pM) suggest that high‐affinity HMP uptake systems are responsible for maintaining low ambient HMP concentrations. We used tritium‐labelled HMP to investigate HMP uptake mechanisms and kinetics in cell cultures of *Candidatus* Pelagibacter st. HTCC7211, a representative of the globally distributed and highly abundant SAR11 clade. A single protein, the sodium solute symporter ThiV, which is conserved across SAR11 genomes, is the likely candidate for HMP transport. Experimental evidence indicated transport specificity for HMP and mechanistically complex, high‐affinity HMP uptake kinetics. Km values ranged from 9.5 pM to 1.2 nM and were dramatically lower when cells were supplied with a carbon source. These results suggest that HMP uptake in HTCC7211 is subject to complex regulation and point to a strategy for high‐affinity uptake of this essential growth factor that can explain natural HMP levels in seawater.

## Graphical abstract

Most marine bacterioplankton have an obligate requirement for Vitamin B1's precursor moiety, HMP, and marine dissolved HMP concentrations are remarkably low (approximately 0.1–50 pM). We hypothesized that HMP uptake systems in marine microbes maintain these low ambient HMP concentrations. Our results suggest that HMP uptake by SAR11 bacteria in cell culture (strain HTCC7211) is subject to complex regulation and could explain natural HMP levels in seawater.

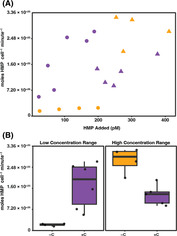

## INTRODUCTION

Thiamin (Vitamin B1; B1) is an essential coenzyme required across all domains of life (Monteverde et al., 2017). Thiamin is required by enzymes throughout both anabolic and catabolic central carbon metabolic processes and amino acid synthesis (Jurgenson et al., 2009; Rapala‐Kozik, 2011). It is synthesized by many prokaryotes in a multi‐step biosynthesis pathway consisting of the separate production of thiazole and pyrimidine moieties, followed by ligation and phosphorylation of the compound (Jurgenson et al., 2009). Thiazole (4‐methyl‐5‐β‐hydroxymethyl thiazole) synthesis in prokaryotes involves 6 genes, eventually leading to the combination of three compounds into the finished thiazole ring (Begley et al., 1999; Jurgenson et al., 2009). Biosynthesis of the pyrimidine moiety, 4‐amino‐5‐hydroxymethyl‐2‐methylpyrimidine (HMP), uses only 2 gene products (Jurgenson et al., 2009) in a one‐step reaction. The rearrangement entails the conversion of 5‐aminoimidazole ribotide (AIR) into HMP‐diphosphate by ThiC (Chatterjee et al., 2010; Jurgenson et al., 2009). Interestingly, this is a distinct biosynthetic pathway from the pathways that generate pyrimidine nucleotides (Garavito et al., 2015). The thiazole ring and HMP are then coupled together (ThiE), and thiamin phosphate kinase (ThiL) adds the final phosphate group to yield thiamin pyrophosphate (TPP), which is the bioactive form of thiamin which can then be utilized by cells (Jurgenson et al., 2009).

Thiamin auxotrophy (obligate requirement caused by the lack of the complete thiamin biosynthesis pathway) is prevalent in marine microbial communities. Early cultivation efforts discovered the existence of thiamin auxotrophy in many marine microorganisms (Hunter & Provasoli, 1964; Provasoli & Carlucci, 1974). These findings have been confirmed by meta‐omics studies, which have demonstrated that most marine microbes lack the necessary genes for complete thiamin biosynthesis (Carini et al., 2014; Paerl, Sundh, et al., 2018; Sañudo‐Wilhelmy et al., 2014). These studies have indicated that thiamin auxotrophy can be caused by the absence of multiple different genes in the thiamin biosynthesis pathway, and that auxotrophs can meet their thiamin demands by utilizing exogenous sources of many different thiamin degradation products and precursor moieties (Carini et al., 2014; Gutowska et al., 2017; McRose et al., 2014; Paerl et al., 2015; Paerl, Bertrand, et al., 2018; Paerl, Sundh, et al., 2018). The most frequent thiamin auxotrophy type found in marine bacterioplankton is the absence of the *thiC* gene, which encodes for the HMP synthesis protein (Paerl, Sundh, et al., 2018).

Dissolved HMP is present in the ocean in picomolar levels (ca. 0.1–50 pM) (Bittner et al., 2024; Bruns et al., 2022; Bruns et al., 2023; Carini et al., 2014; Suffridge et al., 2017; Suffridge et al., 2018; Suffridge et al., 2020). The methods to measure environmental concentrations of HMP (and other thiamin‐related compounds) using liquid chromatography mass spectrometry (LCMS) were recently developed and relatively few published marine dissolved HMP measurements exist (Bittner et al., 2024). Dissolved HMP concentrations in the ocean can vary up to two orders of magnitude across biogeographically distinct regions and depth‐layers (Suffridge et al., 2020). Phytoplankton have been found to release HMP during growth, either through excretion during the exponential phase of growth, or via cell lysis during stationary phase (Carini et al., 2014; Carlucci & Bowes, 1970).

The SAR11 clade of Alphaproteobacteria is the most numerically abundant group of organisms on the planet and SAR11 cells are key players in the global carbon cycle (Giovannoni, 2017; Grote et al., 2012; Morris et al., 2002). SAR11 cells have small, streamlined genomes and have been predicted to oxidize between 6%–37% of gross primary production (White et al., 2019). This is possible, at least in part, due to the use of multifunctional enzymes by SAR11 cells (Clifton et al., 2023; Noell et al., 2021; Noell & Giovannoni, 2019; Wei et al., 2021). Genomic analysis indicates that SAR11 cells almost universally lack the *thiC* gene, meaning they cannot biosynthesize HMP and must acquire this compound from an exogenous source to complete thiamin biosynthesis; exogenous thiamin cannot fulfil their requirements (Carini et al., 2014; Giovannoni, Tripp, et al., 2005). In this study *Candidatus* Pelagibacter st. HTCC7211 was used. This strain was originally isolated from the Sargasso Sea and is a confirmed HMP auxotroph that cannot utilize exogenous thiamin, whose genome contains a putative HMP transport gene, a sodium:solute symporter labelled as *thiV*, as the sole mechanism for meeting its thiamin requirements (Carini et al., 2014; McRose et al., 2014). Sodium:solute symporters are integral membrane proteins that facilitate the transport of both sodium ions and another solute molecule across the cell membrane simultaneously, using the energy stored in the sodium ion gradient (Henriquez et al., 2021). It has been shown that thiamin biosynthetic and acquisition genes, including the *thiV* gene in *Ca.* Pelagibacter, are frequently regulated via a TPP riboswitch which modulates gene expression and protein synthesis when the cellular quota of TPP has been achieved (Atilho et al., 2019; Carini et al., 2014; Donovan et al., 2018; Llavero‐Pasquina et al., 2022; McRose et al., 2014; Meyer et al., 2009; Subki et al., 2020; Winkler et al., 2002).

Here, we describe measurements of HMP uptake by a member of the SAR11 clade, *Candidatus* Pelagibacter st. HTCC7211. We hypothesized that HTCC7211 has a high affinity, multifunctional uptake system for HMP, allowing cells to readily compete for HMP at concentrations measured in the ocean. Due to the role thiamin plays in catalysing key steps in carbon metabolism, we hypothesized that the presence of carbon sources in the experimental system would lead to an increase in uptake rate as cellular requirement for thiamin, and thus HMP, increases. To test these hypotheses, we used tritium labelled HMP to measure uptake by HTCC7211 in cell culture. We aimed to determine the rates and mechanistic details of the machinery used by HTCC7211 to acquire HMP, providing insight into the processes that likely govern HMP availability in the ocean. We aspire to gain a greater understanding of the fine‐scale controls of carbon metabolism in the world's most numerically abundant organism with the goal of elucidating controls on the global carbon cycle.

## EXPERIMENTAL PROCEDURES

### Cell culture conditions

Artificial sea water (ASW) media was prepared for the cultivation of *Ca.* Pelagibacter st. HTCC7211 (hereafter, HTCC7211) as described previously (Carini et al., 2013). Carbon sources were added (pyruvate (100 μM), glycine (50 μM), and methionine (10 μM)) and a vitamin mix (1000 nM Pantothenic Acid, 1 nM Biotin, 1 nM pyrroloquinoline quinone, and 1 nM cyanocobalamin) (Carini et al., 2013). For stock cultures, HMP was added at a final concentration of 1 nM; for cultures used in uptake experiments, this concentration was reduced to 10 pM to grow cells in a starvation state for HMP. This concentration was previously determined as the lowest concentration of HMP at which maximum cell density could be achieved by HTCC7211 (Carini et al., 2014).

Cell density was assessed 3 times per week using flow cytometry. Fluorescent staining using SYBR Green dye and was used as previously described (Tripp, 2008). Briefly, a Millipore Guava flow cytometer was used to analyse cells stained with SYBR Green dye based on cell size, granularity, and SYBR Green fluorescence. The cell counts were automatically calculated using the Cytosoft Express Plus application, then recorded for use during the analysis phase (Tripp, 2008). Cell densities were used to calculate growth rate, which ranged between 0.33 and 0.51 day^−1^ across all experiments, which is consistent with exponential growth of HTCC7211 cultures (Carini et al., 2013). Cells were harvested at a density of 3 × 10^7^–5 × 10^7^ cells mL^−1^. The calculated growth rate and a visual inspection of the cellular growth curve was used to ensure that cells were in exponential growth and had not entered stationary phase at the time of harvest.

### Cell slurry preparation

Centrifugation was used to concentrate and wash HTCC7211 cells. Cells were prepared for centrifugation as described previously (Noell & Giovannoni, 2019). Briefly, experimental replicates were combined and aliquoted equally for initial centrifugation in a Beckman J2‐21 centrifuge at 10°C for 90 min at 12,000 × g. The supernatant was discarded, and pellets were washed by resuspension in 1 mL of unamended (no added carbon sources; −C hereafter) ASW for a second centrifugation in a Beckman–Coulter ultracentrifuge at 10°C for 65 min at 45,000 × g. These steps generated a higher cell density (3 × 10^8^–1 × 10^10^ cells mL^−1^) than is possible in typical HTCC7211 cultures or is found in the environment. From this slurry, 5 mL experimental replicates were produced; triplicate technical replicates were used for each time point. All samples were incubated for 1 h incubation at either 4°C (experimental samples), or on a hot plate at 80°C (heat‐killed negative control cultures). All incubations were conducted in the dark to prevent energy production by rhodopsins (Giovannoni, Bibbs, et al., 2005; Steindler et al., 2011). Cell density in the slurry was measured as described above, preceded by serial dilution.

### Tritium labelled HMP uptake

HMP uptake by HTCC7211 was measured by adding tritiated HMP (^3^H‐HMP) into the concentrated cell slurry, killing the cells at specified time points after the ^3^H‐HMP addition, and measurement of ^3^H on a liquid scintillation counter, following a method adapted from previous studies of SAR11 uptake (Noell & Giovannoni, 2019). The ^3^H‐HMP was obtained via custom synthesis by American Radiolabeled Chemicals (St. Louis, Missouri) at a concentration of 250 μCi mL^−1^ and a specific activity of 60 Ci mmol^−1^. This stock was diluted 1:250 to a final concentration of 1 mM ^3^H‐HMP and stored at −20°C. Uptake was measured at 0, 5, 10, and 60 min to determine the rate of uptake and maximum intracellular concentration reached. Replicate samples were brought back to room temperature by incubating for 5 min at 35°C. ^3^H‐HMP was spiked into experimental replicates and inverted to mix, followed by placing in a 25°C water bath for incubation until the specified time point was reached to allow peak metabolic activity (Noell & Giovannoni, 2019). At the specified time points, 100 μL of 500 mM sodium azide was added to kill the cells, and replicates (technical triplicates) were immediately placed on ice. Triplicate heat‐killed controls were incubated for 60 min. HMP uptake in all heat‐killed controls were below the readings for the 0 min samples. Experimental replicates were vacuum filtered onto Omnipore™ 0.1 μm PFTE membrane filters and washed with ASW media to remove residual extracellular ^3^H‐HMP. The filters were placed into scintillation vials with 5 mL scintillation fluid, shaken vigorously, and placed in the dark overnight before analysis using a Beckman Coulter LS 6500 Multi‐Purpose liquid scintillation counter.

HMP uptake was also measured in the presence of carbon sources (+C) by adding 100 μM pyruvate, 50 μM glycine, 10 μM methionine, and vitamin mixture (as described above, but excluding HMP) to the cell slurry prior to uptake assay and analysis. Experiments followed the same procedure described above.

**FIGURE 1:**
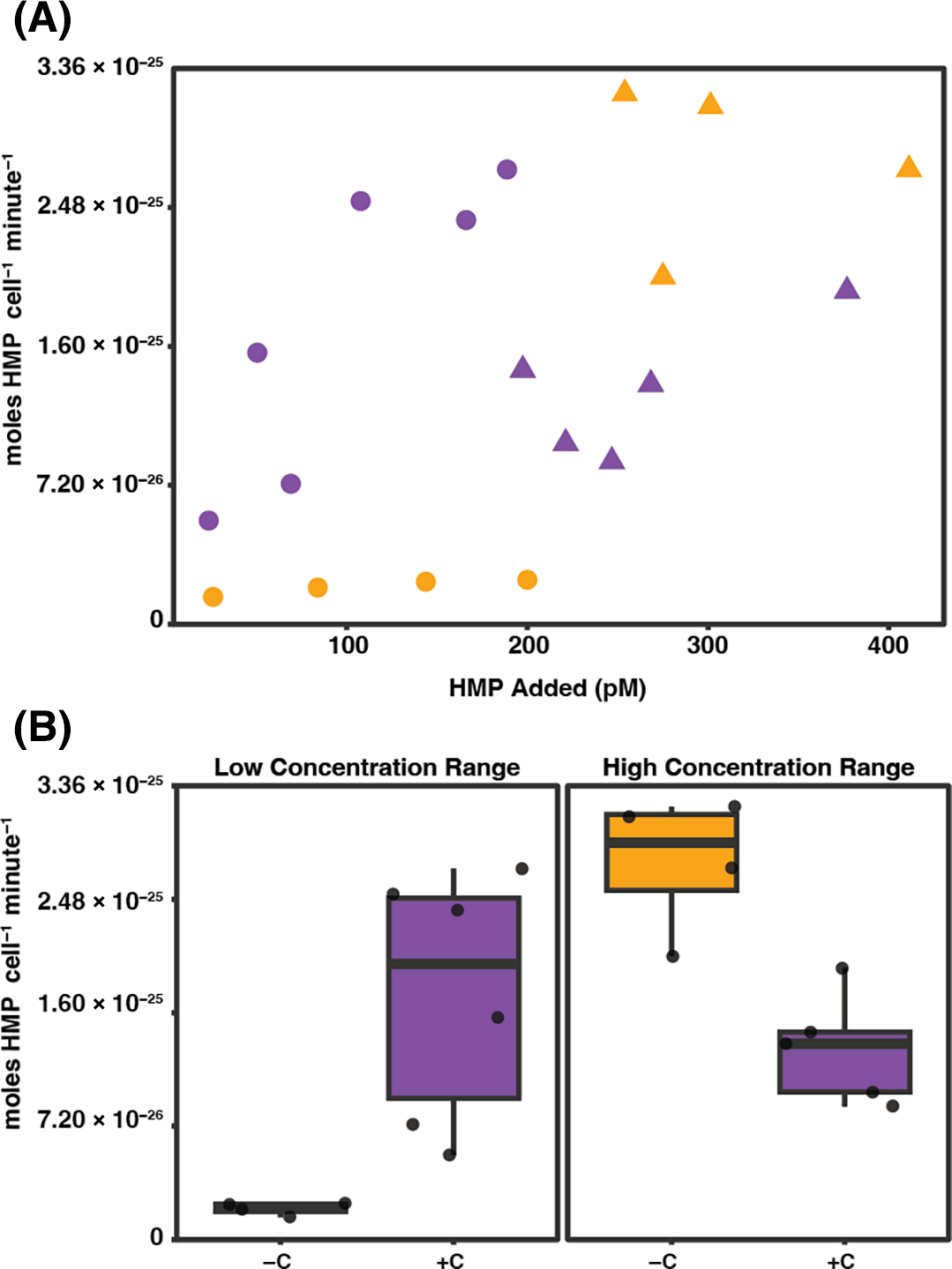
HMP uptake by *Ca.* Pelagibacter st. HTCC7211. (A) The rate of HMP uptake (moles HMP cell^−1^ min^−1^) by SAR11 strain HTCC7211 cells at specific concentrations of added HMP (pM). Circles represent the low concentration range and triangles represent the high concentration range. Yellow indicates experiments with −C and purple indicates experiments with +C. (B) Boxplots show the range of HMP uptake rates (moles HMP cell^−1^ min^−1^) observed in the low and high range of HMP additions in both −C (yellow) and +C (purple) treatments.

Within the −C experiments, we observed HMP uptake rates to be 20‐fold greater at added HMP concentrations greater than 250 pM HMP compared with concentrations lower than 250 pM (Figure 1). We therefore created low HMP (<250 pM HMP) and a high HMP (>250 pM HMP) concentration bins for experimental analyses. Representative low and high HMP concentrations (55 and 285 pM, respectively) were used in induction and competitive inhibition experiments.

### Induction experiments

HMP uptake was analysed at greater resolution during the first 8 min of uptake to identify the presence of uptake induction. Time points included 0 min, 30 s, 1, 2, 4, 8, and 60 min. Representative concentrations of both low and high concentration ranges of HMP were analysed (55 and 285 pM), and both concentrations were analysed both without (−C) and with (+C) carbon source addition for a total of 4 induction experiments.

### Competitive inhibition experiments

HMP uptake was analysed in the presence of other pyrimidine compounds to observe for potential competitive inhibition: cytosine, thymine, uracil, and known thiamin degradation product 4‐amino‐5‐aminomethyl‐2methylpyrimidine (AmMP). A total of four experiments were conducted, using both low and high concentration range representatives of HMP (55 and 285 pM respectively), both −C and + C. Experiments were conducted as previously described above, but with all replicates incubated in the presence of ^3^H‐HMP for 10 min. Competitors were added to experimental replicates immediately before ^3^H‐HMP addition in excess, with a ratio of 1:4 HMP:competitor at 285 pM HMP, and 1:20 HMP:competitor at 55 pM HMP. Water was used as a negative control. Glucose was used as a positive control to control for molecular dilution as HTCC7211 is unable to assimilate glucose.

### Data analysis

The uptake rate for HMP into HTCC7211 was calculated individually for each experiment and analysed for statistical significance. Using the total counts per minute (CPM), decay constant and specific activity of ^3^H‐HMP, CPM was converted to moles of total HMP. The cell counts obtained previously were used to normalize this measurement to a per cell basis (moles HMP cell^−1^). These values were plotted against time and fit with a linear regression (base R function) over the first 10 min to determine uptake rate. Adjusted *R*‐squared values across all uptake experiments ranged from 0.72 to 0.99 with a mean value of 0.95. The linear model fit was statistically significant for every experiment (*p*‐value <0.05). The total HMP concentration added in each experiment was additionally calculated using the scintillation counts from the positive controls, recorded H#, decay constant, and specific activity of tritium. H#, or transformed Spectral Index of External Standard (tSIE), describes the amount of quenching present in the sample; quenching is anything, which interferes with the conversion of decay energy emitted from the sample vial into light photons (L'Annunziata et al., 2020). Non‐linear regression was used to fit the Michaelis–Menten equation to a plot of uptake rate (V) versus HMP concentration (S) using the nls function in R, as has previously been reported (Greco & Hakala, 1979).

To analyse statistically significant effects of carbon on uptake rates over a range of HMP concentrations, a linear model was applied and *p*‐values recorded. The appropriateness of this model was determined using ggdensity and ggqqplot, observing for skewness. A Shapiro test was also conducted for quality control, using the *p*‐value to determine normal distribution of data, with *p*‐values above 0.05 indicating normal distribution and error within the data. The linear model was used to compare uptake in the presence and absence of carbon within the low and high concentration ranges separately, and a final Breusch–Pagan Test to check for heteroscedasticity.

To analyse competitive inhibition of uptake by other pyrimidine compounds, a Dunnett's test was performed comparing all competitor treatments back to the water addition control. The 10‐min intracellular concentration of HMP (moles HMP cell^−1^) for each treatment was averaged and calculated as a percent of the water control average value. These percentages were then used to analyse for statistically significant effects (*p*‐value <0.05) of the competitors on the uptake of HMP by SAR11 HTCC7211 cells using the Dunnett's test.

### Bioinformatics analysis

Putative thiamin‐related enzymes and transporters were queried from the Eggnog v6 and Uniprot databases (Hernandez‐Plaza et al., 2023; UniProt, 2023). Both non‐specific (e. g. thiamin transporter, HMP transporter) and specific (e. g CytX, ThiBPQ) transporters were used as text searches against the database to identify any potential candidates of SAR11 HMP importer function (See Table S1 for specific details). The ThiV/SSSP protein (SAR11_0811 from HTCC1062) described by Carini et al. (2014) was identified, along with a putative CytX homologue called PucI that was previously unexamined (SAR11_0868 in HTCC1062) (Carini et al., 2014).

Orthologs of the SAR11_0811 protein (LCOG0591 in the Eggnog6 database) were selected to encompass well‐characterized prokaryotic and eukaryotic sodium‐solute symporters, including the SiaT sialic acid, PanF pantothenate, and OpuE and PutP proline symporters. Several HMP/Thiamin symporters were also chosen, including SSSF, SSSQ and SSSP proteins. Crystallized structure (for SiaT) and predicted tertiary structure (for others, pre‐computed using AlphaFold) were used as input to the mTM‐align program to produce a multiple sequence alignment and to generate a TM‐score matrix (Dong et al., 2018). The multiple sequence alignment was manually examined and compared with the previously described crystallized structure for transmembrane domains and amino acid residues essential for substrate binding (Henriquez et al., 2021; Wahlgren et al., 2018).

A similar approach was taken for analysis of the SAR11_0868 protein (LCOG1953 in the Eggnog6 database), wherein the well‐characterized PLUTO protein from *Arabidopsis thaliana* and the crystallized structure of HyuP/Mhp1 from *Microbacterium liquefaciens* were used as foci for comparison, along with several other nucleobase transporters from the same orthologous group (See Table S2 for more details).

## RESULTS

### Tritium labelled HMP uptake

We measured uptake of HMP by HTCC7211 using ^3^H‐HMP in the absence of exogenous carbon sources (−C) at total HMP concentrations between 26 and 410 pM (Figure 1) and observed complex, atypical uptake kinetics. The concentrations of HMP used for experimentation are between one and two orders of magnitude greater than observed marine dissolved HMP concentrations (Bittner et al., 2024; Bruns et al., 2022; Bruns et al., 2023; Carini et al., 2014; Suffridge et al., 2017; Suffridge et al., 2018; Suffridge et al., 2020). However, the cell densities in our experiments are also one to two orders of magnitude greater than what is found in the environment (Morris et al., 2002). Therefore, on a basis of standing stock HMP per cell, the concentrations of HMP used are equivalent to observed marine dissolved HMP concentrations. At HMP concentrations ≤250 pM we found uptake rates below 1.22 × 10^−26^ moles HMP cell^−1^ min^−1^ (Table 1). In contrast, we observed a greater than 20‐fold increase in HMP uptake rates at HMP concentrations ≥250 pM, where rates reached a maximum of 3.20 × 10^−25^ moles HMP cell^−1^ min^−1^ (Table 1). To aid in data interpretation and experimental design we created low HMP concentration range and high HMP concentration range bins separated at 250 pM added HMP. The mean rate of uptake in the low concentration range was 7.96 × 10^−27^ moles HMP cell^−1^ min^−1^, while in the high concentration range, the mean rate was 2.77 × 10^−25^ moles HMP cell^−1^ min^−1^ (Figure 1), and these values significantly differed (*p*‐value = 0.004; linear model).

**TABLE 1:** HMP Uptake Rates (moles HMP cell^−1^ min^−1^) at specific concentrations of added HMP (pM) in both −C and +C treatments.

Total HMP added (pM)	Uptake rate (moles HMP cell^−1^ min^−1^)	Carbon
26	1.37 × 10^−27^	−DOC
84	7.30 × 10^−27^	−DOC
143.8	1.10 × 10^−26^	−DOC
200	1.22 × 10^−26^	−DOC
253.9	3.20 × 10^−25^	−DOC
275.1	2.04 × 10^−25^	−DOC
301.4	3.12 × 10^−25^	−DOC
411.2	2.72 × 10^−25^	−DOC
23.6	4.97 × 10^−26^	+DOC
50.5	1.56 × 10^−25^	+DOC
69.1	7.30 × 10^−26^	+DOC
107.7	2.52 × 10^−25^	+DOC
166.1	2.40 × 10^−25^	+DOC
188.7	2.72 × 10^−25^	+DOC
197.5	1.45 × 10^−25^	+DOC
221.2	9.86 × 10^−26^	+DOC
246.8	8.72 × 10^−26^	+DOC
268.3	1.36 × 10^−25^	+DOC
376.9	1.95 × 10^−25^	+DOC

We observed a biphasic model of uptake in the −C treatments, which indicated that calculations of the Michaelis–Menten kinetics parameters, *K*_m_ and *V*_max_ should be determined separately for each concentration range (Table 2). When these calculations were attempted for the low concentration range using a nonlinear least‐squares method, the model fit the data well (*R* ^2^ = 0.97), with calculated *V*_max_ and *K*_m_ values of 3.07 × 10^−26^ moles HMP cell^−1^ min^−1^ and 119 pM HMP, respectively, although we caution that this calculation is only based on four data points. For the high concentration range the fit was poor (*R* ^2^ = 0.25), with calculated *V*_max_ and *K*_m_ values of 2.49 × 10^−24^ moles HMP cell^−1^ min^−1^ and 1156 pM HMP, respectively. The poor fit for the high concentration range data indicates that further validation of these kinetic parameters is needed.

**TABLE 2:** Calculated kinetic parameters for uptake data of HMP by HTCC7211.

Treatment/range	*K*_m_ (pM)	*V*_max_ (moles HMP cell^−1^ min^−1^)	Nonlinear least‐squares fit, *R* ^2^
−C, low concentration	119	3.07 × 10^−26^	0.97
−C, high concentration	1156	2.49 × 10 ^−24^	0.25
+C, all concentrations	9.5	1.88 × 10^−25^	0.17

*Note*: A non‐linear least squares regression was used to fit the Michaelis Menten equation to the data. The −C uptake was split into two concentration ranges given the clear plateau in uptake rates at the high end of the low concentration rate (Figure 1). The final data point from the low concentration range was also included in the high concentration range.

### Carbon addition experiments

We next tested our hypothesis that increased dissolved organic carbon concentrations would result in increased cellular requirements for vitamin B1 and thus lead to increased uptake rates of HMP. To test this hypothesis, we repeated our previous ^3^H‐HMP uptake experiments with the addition of the same carbon sources and concentrations used in the HTCC7211 growth medium (pyruvate, glycine, methionine, and the vitamin mixture, not including HMP) (Carini et al., 2013). In the low concentration range of uptake, the +C treatment resulted in uptake rates that were much closer to those measured in the high concentration range than those −C (mean of 1.74 × 10^−25^ moles HMP cell^−1^ min^−1^) (Table 1, Figure 1), indicating a clear influence of carbon source availability on HMP uptake, even when its concentrations are low. Indeed, adding carbon sources statistically significantly increased uptake rates above those measured in the low concentration range −C experiments (*p*‐value = 0.003; linear model). In contrast, in the high concentration range +C, uptake rates were significantly lower than −C (average of 1.32 × 10^−25^ moles HMP cell^−1^ min^−1^ + C; *p*‐value = 0.006; linear model) (Table 1, Figure 1). When we repeated kinetic parameter calculations for all the uptake data from the +C treatments, we found a poor fit (*R* ^2^ = 0.17) and calculated *V*_max_ and *K*_m_ values of 1.88 × 10^−25^ moles HMP cell^−1^ min^−1^ and 9.5 pM HMP, respectively (Table 2).

### Induction experiments

**FIGURE 2:**
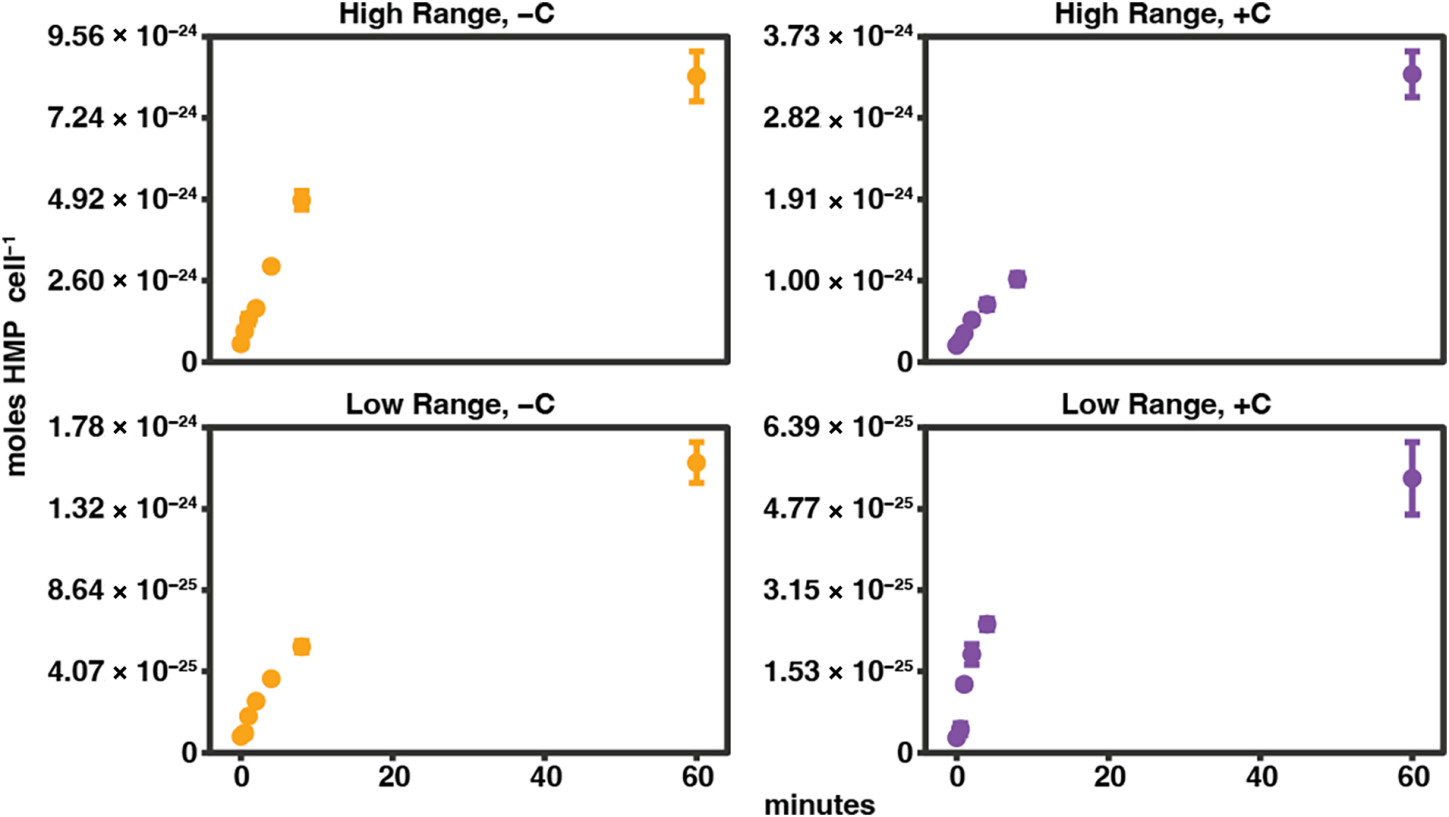
Induction of HMP Uptake in *Ca.* Pelagibacter st. HTCC7211 was not observed. HMP uptake (moles HMP cell^−1^) was measured over 60 min. Experiments were conducted at the low (55 pM) and high (285 pM) ranges of added HMP and were tested with both −C (yellow) and +C (purple) treatments.

**FIGURE 3:**
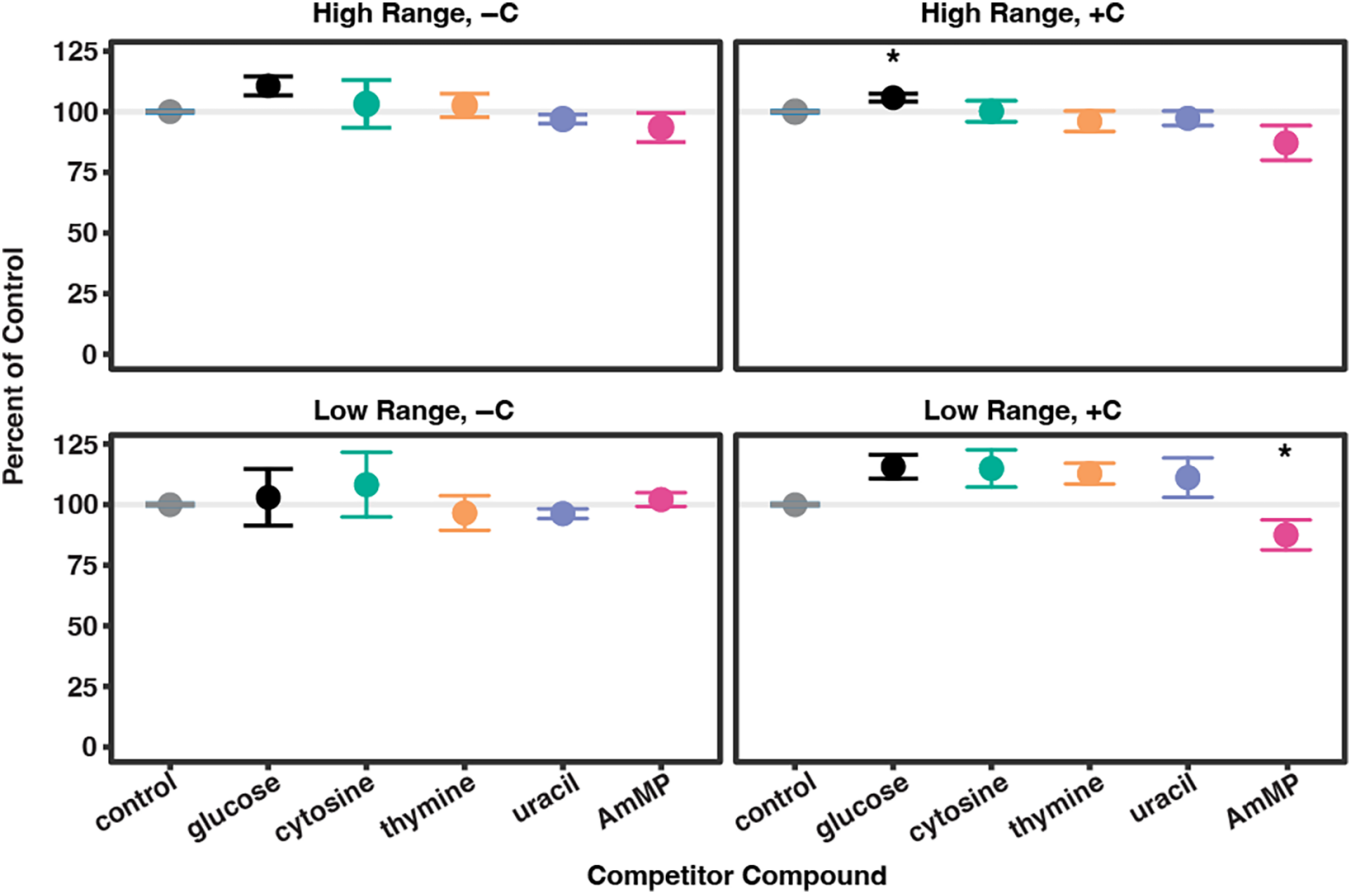
Competitive inhibition of HMP uptake in *Ca.* Pelagibacter st. HTCC7211. Competitive inhibition of HMP uptake was assessed using four other structurally similar pyrimidine compounds: Cytosine, thymine, uracil and AmMP. Cells were incubated in the presence of each compound at both the low (55 pM) and high (285 pM) range of added HMP, and in both the presence and absence of added carbon sources. Compounds were provided in excess, 1:20 HMP:Competitor in the low range and 1:4 HMP:Competitor in the high range. Asterisks indicate significant differences from the control.

To determine whether genetic induction played a role in the observed 20‐fold increase in uptake rates between the low and high concentration ranges both −C and +C, we explored whether HMP uptake is regulated by the presence of HMP in any manner (Figure 2). To accomplish this, we measured uptake of HMP at high resolution within the first 10 min of uptake at one representative concentration from each of the two concentration ranges: 55 and 285 pM HMP. If transcriptional regulation were occurring, we would expect to see a sharp inflection of HMP uptake within 1–3 min of the experiment (Noell et al., 2023). In both low concentration range treatments, we did observe a spike (3–4× increase) in uptake rate from 0.5 to 1 min, with uptake rates then decreasing after 1 min to similar levels measured from 0 to 0.5 min (Figure 3, Table 3). In the high concentration range treatments, uptake rates remained relatively constant during the entire time‐course. In all treatments, the uptake rate did not shift dramatically between the last two time points (8–60 min). Due to the lack of a consistent increase in HMP uptake found during these higher resolution analyses, we determined there is likely no transcriptional regulation of the HMP transport system in HTCC7211.

**TABLE 3:** Induction of HMP uptake (moles HMP cell^−1^) was assessed over 60 min at both the low (55 pM) and high (285 pM) ranges of added HMP, and were tested with both −C and +C treatments.

	Low range of (HMP)		High range of (HMP)	
Minutes	20 pM, −DOC added	20 pM, +DOC	100 pM, −DOC	100 pM, +DOC
0.5	3.65 × 10^−26^	3.44 × 10^−26^	7.26 × 10^−25^	1.03 × 10^−25^
1	1.90 × 10^−25^	1.78 × 10^−25^	6.75 × 10^−25^	1.64 × 10^−25^
2	8.45 × 10^−26^	5.97 × 10^−26^	3.10 × 10^−25^	1.50 × 10^−25^
4	6.30 × 10^−26^	3.00 × 10^−26^	6.00 × 10^−25^	8.70 × 10^−26^
8	4.50 × 10^−26^		4.71 × 10^−25^	7.13 × 10^−26^
60	1.99 × 10^−26^	5.19 × 10^−27^	6.81 × 10^−26^	4.41 × 10^−26^

### Competitive inhibition experiments

Finally, we asked whether the HMP transport system in HTCC7211 is multifunctional or promiscuous (i.e., capable of transporting multiple compounds in addition to HMP). We used the same representative HMP concentrations (55 pM and 285 pM HMP) from the low and high concentration ranges of uptake as before, with either −C or + C. To these four treatments, we added one of four different competitor compounds (cytosine, thymine, uracil or AmMP) along with the ^3^H‐HMP and measured HMP uptake after 10 min. The four competitor compounds were selected due to their similar pyrimidine structure to HMP, as well as biological relevance to the cell. Cytosine, thymine and uracil are nucleotides and AmMP (4‐amino‐5‐aminomethyl‐2‐methylpyrimidine) is a known thiamin breakdown product only differing from HMP by a single functional group (Gutowska et al., 2017). If a competitor compound was being transported by the same transport system as HMP, we would expect to see a significant decrease in HMP uptake in that treatment compared with the negative control. We observed statistically significant differences in HMP uptake under 2 conditions using a Dunnett's test. In the low HMP concentration range +C experiment, the glucose control was found to be significantly different from the water control (*p*‐value = 0.015). Additionally, in the high HMP concentration range +C experiment, the AmMP treatment was found to be statistically significantly different from the water control (*p*‐value = 0.005). However, the magnitude of the influence on HMP uptake was small in both cases (<15% of the control treatment), indicating that any competitive inhibition or enhancement taking place is minor in nature and the HMP transporter is quite specific for HMP. No other significant differences in HMP uptake were found, despite that the competitor compounds being added in excess of HMP (1:20 and 1:4 ratio of HMP:competitor in the low and high concentration ranges, respectively) (Figure 3).

### Computational analysis of putative HMP import protein

One possible explanation for the observed biphasic model of HMP uptake is that the HTCC7211 genome encodes multiple HMP import systems. To explore this hypothesis further, we leveraged the manually curated Swiss‐Prot database in Uniprot and the pre‐computed ortholog database Eggnog 6 (Hernandez‐Plaza et al., 2023; UniProt, 2023). We ruled out presence of several characterized HMP/Thiamin transporter systems but did identify a homologue of the PLUTO transporter (called CytX in bacteria) encoded on the *Arabidopsis thaliana* plastid genome that was recently characterized as an HMP importer (Beaudoin et al., 2018) (Table S1). The Eggnog 6 orthogroup LCOG1953 includes CytX from SAR11 (SAR11_0868 in HTCC1062, PB7211_748 in HTCC7211) along with several other nucleobase importers (Table S2). While CytX from SAR11 has the highest tertiary alignment score with PLUTO from *Arabidopsis thaliana*, it does not share conserved substrate‐binding residues with PLUTO when compared to the solved crystal structure of HyuP/Mhp1 from *Microbacterium liquefaciens* (Table S3, alignment in File S1). Therefore, the specificity of the SAR11 CytX protein for HMP or other nucleobases is yet to be determined.

**FIGURE 4:**
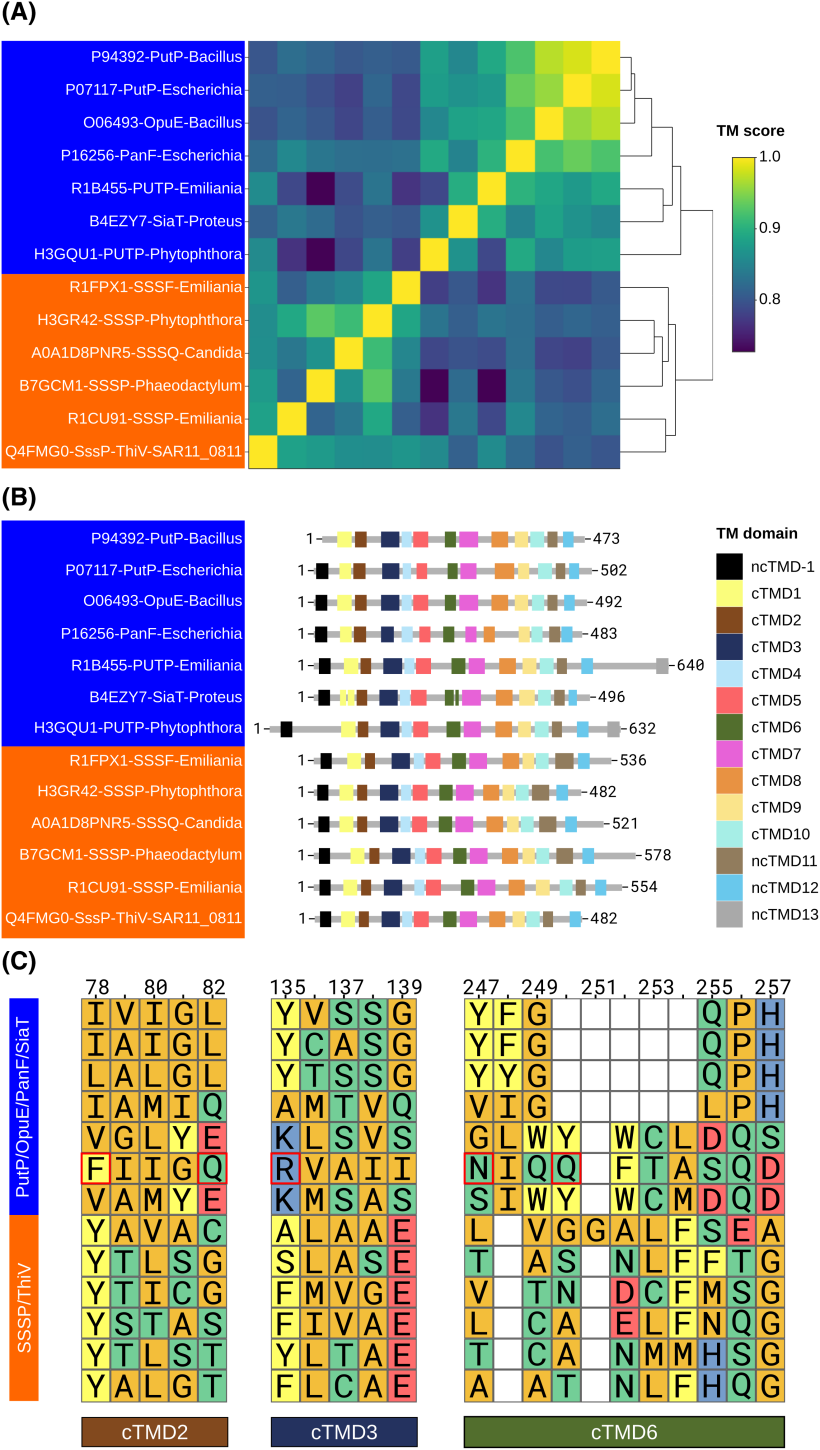
SAR11 encodes a sodium:HMP symporter. (A) Heatmap of pairwise TM scores calculated using tertiary alignment of predicted tertiary structure and experimentally derived tertiary structure of SiaT from *Proteus mirabilis*. The SAR11 protein ThiV clusters with Thiamin/HMP symporters from Eukaryotes rather than symporters characterized in Prokaryotes. Scores are clustered based on the Ward. D2 algorithm and the dendrogram on the right depict this clustering; TM scores are coloured as shown in the legend, with higher scores indicating more similar tertiary structure. (B) Domain structure of selected sodium: Solute symporters. All symporters have at minimum, the conserved transmembrane domains (cTMD) 1–10. Non‐conserved transmembrane domains (ncTMD‐1, ncTMD11‐13) are present in some symporters as well. All symporters included in this analysis are predicted to be functional. Transmembrane domains are coloured based on the legend as shown on the right. Transmembrane domains are predicted based on the multiple sequence alignment to the structurally characterized SiaT protein. (C) Multiple sequence alignment of substrate binding domains. Selected positions (positions in the SiaT protein are numbered at the top) of the multiple sequence alignment of cTMD2, cTMD3 and cTMD6 are shown. Characterized substrate binding positions from SiaT are boxed in red. HMP/Thiamin symporters share a unique tyrosine residue at position 78 in cTMD2, aspartic acid at position 139 in cTMD3, and all but one shared a phenylalanine at position 254 in cTMD6. These residues correspond to positions either at (cTMD2) or near (cTMD3, cTMD6) the substrate‐binding positions in SiaT. Colour‐coding is based upon amino acid side chain, with Red = Carboxyl, Orange = Non‐polar, Blue = Amine, Green = Polar, Yellow = Aromatic.

As reported in Carini et al., 2014, ThiV (SAR11_0811), a sodium: solute symporter encoded in HTCC1062 and other members of the Pelagibacterales order, is the best candidate for putative HMP import. However, the best characterized prokaryotic proteins that are homologues of the ThiV protein are annotated as proline symporters in *Escherichia coli* and *Bacillus* spp. (Table S4). In order to provide evidence for the functional identify of the ThiV protein, and eliminate support for the putative proline function, we aligned tertiary protein structures as predicted by Alphafold to the experimentally verified tertiary structure for the SiaT Sialic Acid symporter from *Proteus mirabilis* (Figure 4; Table S5; File S2) (Henriquez et al., 2021; Wahlgren et al., 2018). We found that, indeed, the SAR11_0811 protein encoded in HTCC1062 (PB7211_402 in HTCC7211) clusters with the known Eukaryotic HMP/thiamin symporters SSSP and SSSQ rather than the various prokaryotic sodium: solute symporters based on TM‐Score (Figure 4A).

All examples of the HMP/thiamin symporters, including ThiV from SAR11 strains, encode at least the 10 conserved sodium‐solute symporter transmembrane domains (cTMD1‐10) and other additional transmembrane domains (ncTMD‐1, ncTMD11‐13; Figure 4B; Table S6) (Wahlgren et al., 2018). Encoding these transmembrane domains provides support for functional sodium: substrate symport into the cells.

Finally, with respect to functional specificity, we identified several unique and conserved residues in the multiple sequence alignment in the putative HMP:thiamin transport proteins (Figure 4C). A well‐conserved tyrosine residue is found at position 78, along with several polar amino acids just downstream in the conserved transmembrane domain 2 (cTMD2). We find a well‐conserved acidic residue at position 139, several residues away from the substrate‐binding residue of 135 in SiaT (Figure 4C). Lastly, the substrate‐binding residues of cTMD6 are more diverse than the other two domains, with gaps in the alignment, which indicates less conservation or putatively an indel at this location in the protein. A conserved phenylalanine at position 254, followed by a histidine and/or polar residues is unique to the HMP/thiamin symporters (Figure 4C). The conservation of residues at binding sites provides support for the hypothesis that the ThiV protein in SAR11 is indeed an HMP transport protein, despite the best functional characterization of these proteins coming from Eukaryotic organisms.

## DISCUSSION

Our data provide the first recorded uptake rate measurements of HMP into SAR11 cells and are strong experimental evidence that HTCC7211 can assimilate HMP from the marine dissolved pool as previously reported based on the growth factor requirements of cultures (Carini et al., 2014). The high affinity uptake kinetics of HMP found in HTCC7211, where Km values range from 9.5 pM to 1.2 nM (Table 2), suggest that the concentrations of the global standing stock of dissolved HMP, which have been a reported range between ca. 0.1 and 50 pM (Bittner et al., 2024; Bruns et al., 2022; Bruns et al., 2023; Carini et al., 2014; Suffridge et al., 2017; Suffridge et al., 2018; Suffridge et al., 2020), could be controlled by microbial uptake mechanisms. The high affinity uptake of dissolved HMP is in accord with previous observations, which showed that SAR11 cells have high affinity uptake systems for high priority compounds (Noell & Giovannoni, 2019). For example, past studies have measured whole‐cell *K*_s_ values of 0.89 and 1.85 nM for *Ca.* Pelagibacter st. HTCC7211 and *Ca.* Pelagibacter ubique st. HTCC1062, respectively (Noell & Giovannoni, 2019). For comparison, other cells have had measured whole‐cell *K*_s_ values larger (i. e., lower affinity) than SAR11 for similar compounds: for *E. coli* for thiamin, measured *K*_s_ was 6.9 nM (Yamada & Kawasaki, 1980) while for *Bacillus cereus*, measured *K*_s_ was 20 nM (Tobüren‐Bots & Hagedorn, 1977).

We observed atypical HMP uptake kinetics in HTCC7211. Biphasic uptake was observed in the −C treatments, while this pattern was not seen in +C treatments (Figure 1). Within the low concentration range, HMP uptake was observed to be significantly higher in +C treatments over −C treatments (*p*‐value = 0.003; linear model). The increase in HMP uptake rate when exogenous carbon sources are available could be tied to vitamin B1's role in catalysing key steps in central carbon metabolism. It is reasonable to hypothesize that increased cellular metabolic rates could lead to increased vitamin B1 requirements and thus higher rates of HMP uptake to meet those demands.

Biphasic uptake patterns, such as those that we observed in the −C treatments, are not unprecedented; generally, they are attributed to either two transporter systems with distinct kinetic properties simultaneously being active, or a single transporter system that is transcriptionally inducible. Past examples of biphasic uptake in microorganisms include uptake of Vitamin B_12_ into the mitochondrion of the freshwater mixotrophic algae *Euglena gracilis* (Sarhan et al., 1980), flagellate algae *Ochromonas malhamensis* (Bradbeer, 1971), *Escherichia coli* (Bradbeer & Woodrow, 1976), amino acid uptake by *E. coli* (Robbins James & Oxender Dale, 1973) and glucose uptake in *Streptococcus bovis* (Russell, 1990) have also been observed to be biphasic. In most of these instances (*Euglena* mitochondria, *S. bovis*, and *E. coli*), this biphasic uptake was attributed to two separate transport systems, one energy‐independent one energy‐dependent (Bradbeer, 1971; Robbins James & Oxender Dale, 1973; Watanabe et al., 1993).

We used bioinformatics analysis of publicly available genomes to search for a second putative HMP transporter and thiamin‐associated regulatory elements in HTCC7211. We found that all phylogroups of SAR11 encode for at least one HMP/thiamin transporter (Figure 4). Groups 1 (including HTCC7211), 2, and 3 encode a *ThiV/SSSP* homologue (Figure 4). While *ThiV/SSSP* homologues have been shown to uptake thiamin and HMP in other organisms, this is the first direct evidence that HMP is transported by this system. All SAR11 phylogroups encoded a putative TPP riboswitch upstream of *ThiV/SSSP*. Tandem TPP‐HMP riboswitches have been reported in other organisms, but based on our analysis we find it unlikely that this system exists in SAR11 (Atilho et al., 2019). The presumed function of the TPP‐HMP riboswitches is repression of expression of downstream genes when sufficient intracellular TPP is present, but in at least one case (Llavero‐Pasquina et al., 2022) this could not be confirmed, leaving doubt about mechanism of action of these riboswitches. There was no clear evidence of a second HMP or thiamin transporter in HTCC7211 (Figure 4); however, a homologue of PLUTO/CytX, which was recently confirmed to transport HMP in *Arabidopsis* (Beaudoin et al., 2018), was detected in the HTCC7211 genome, but could not reliably be assigned a function because it lacked conserved substrate‐binding residues.

Our results suggest that HTCC7211 cells possess only a single constitutively expressed transport system for HMP uptake (ThiV), that is likely functional and specific for HMP. If HMP transport was transcriptionally regulated by the presence of HMP, we would expect to see a steady increase in uptake rates with increased time of exposure to HMP as the genes for HMP transport are expressed, as observed previously for other bacteria and other inducer compounds (Noell et al., 2023). We did not observe this in any of our treatments (Figure 2). However, we did observe a 3–4× increase in HMP uptake in the low concentration range treatments between 0.5 and 1 min, but then uptake rates dropped again to roughly half this rate and steadily decreased for the remainder of the 60‐min experiment (Figure 3, Table 3). This same result was previously observed for HTCC7211 with L‐alanine uptake, with the spike in uptake rates being attributed to post‐transcriptional regulation as a way to maximize uptake during encounters with ephemeral nutrient patches (Noell et al., 2023). It could be that a similar post‐transcriptional regulatory process is involved in regulating expression or activity of the HMP transporter gene in HTCC7211.

Our results indicated that HMP transport was immediately active in the cultures but attenuated over the course of an hour of exposure to HMP in all treatments (Figure 3; Table 3). A plausible explanation for this pattern is the TPP riboswitch located upstream of the *thiV* sodium: solute symporter gene (Carini et al., 2014; McRose et al., 2014). This riboswitch is proposed to turn off transcription of *thiV* when the cellular quota of TPP is achieved (Carini et al., 2014); our findings are consistent with this hypothesis, but further research is needed to confirm the ligand specificity and activity of this riboswitch.

HMP auxotrophy is the most common form of thiamin auxotrophy, impacting many marine microbes, including SAR11, where this form of auxotrophy was first observed in free‐living bacteria (Carini et al., 2014; Paerl, Sundh, et al., 2018). This observation was concomitant with the rising awareness of interspecies interactions in microbial ecology, which has been underscored by discoveries of highly specific mechanisms of interaction (e.g. Ligand cross‐feeding resolves bacterial vitamin B12 auxotrophies (Wienhausen et al., 2022)). Findings we report here show that the highly specific SAR11 growth requirement for HMP is met by a complexly regulated, high affinity, high‐specificity transport system. In contrast, previous work has underscored a role in SAR11 cells for transporter multifunctionality, which is thought to be favoured by streamlining selection because it preserves functionality while reducing the genome size (Giovannoni, Tripp, et al., 2005; Noell et al., 2021; Noell & Giovannoni, 2019).

Across SAR11 biology, there is little evidence that specific interactions have evolved that reduce genome complexity. For example, their unusual growth requirements for reduced sulfur and precursors of glycine biosynthesis are met by broad range of environmentally available compounds (Giovannoni, 2017). To explain the broadly conserved focus of SAR11 on a single thiamin precursor, HMP, Carini et al. (2014) showed that this hydrophobic compound appears to be a common, leaky product of thiamin production by autotrophic cells and is thus widely available. Complex regulation and specificity of HMP uptake that we describe could in part be due to cytotoxic properties of HMP at high concentrations, which have been described previously, and may drive the evolution of mechanisms that control intracellular concentrations (Garavito et al., 2015; Haughton & King, 1958; Rogers, 1970). Additionally, it is known that toxic HMP derivatives are produced by microbes, which could form the basis of allelopathic cellular interactions that favour highly specific uptake mechanisms in HMP auxotrophs (Cooper et al., 2014; Kraft & Angert, 2017; Reddick et al., 2001; Zilles et al., 2000).

The SAR11 transport affinities for HMP that we report are ecologically significant because they lie in the range of observed variation in ocean dissolved HMP concentrations, and thus provide an explanation for why HMP is most often found at picomolar concentrations and does not accumulate in the dissolved pool. Because of their small size, high substrate affinities and natural abundances, SAR11 cells are positioned to control ambient concentrations of many common metabolites. They have previously been shown to have the highest affinity measured for glycine betaine that are in the range of measured ocean glycine betaine concentrations (Noell & Giovannoni, 2019). We are therefore able to postulate that SAR11 cells could dominate ocean uptake of HMP and determine its ambient background concentrations. Further research may help us understand why this complicated and specialized transport system became a conserved feature of SAR11 metabolism.

## AUTHOR CONTRIBUTIONS

**Elizabeth Brennan:** Methodology; data curation; investigation; formal analysis; visualization; writing – original draft; writing – review and editing. **Stephen Noell:** Conceptualization; methodology; data curation; investigation; writing – review and editing; writing – original draft. **Edward W. Davis II:** Investigation; methodology; software; formal analysis; writing – review and editing. **Stephen J. Giovannoni:** Conceptualization; funding acquisition; writing – review and editing; project administration; resources; supervision. **Christopher P. Suffridge:** Conceptualization; methodology; data curation; investigation; validation; formal analysis; supervision; visualization; project administration; writing – original draft; writing – review and editing.

## CONFLICT OF INTEREST STATEMENT

The authors declare no conflicts of interest.

## Supporting information


**Table S1.** Presence/absence of putative HMP/Thiamin transport system components. Reference HMP/Thiamin transport system proteins uniprot and Eggnog6 accessions are shown, along with references where the proteins were previously characterized. Presence/absence and locus tags for SAR11 HTCC7211 and HTCC1062 are shown.
**Table S2.** Included CytX/PLUTO homologues from the Eggnog 6 orthogroup LCOG1953. Accessions and organism names for the CytX homologues, along with the reason for inclusion of each protein, are shown.
**Table S3.** Pairwise TM scores of selected PLUTO/CytX homologues determined by mTM‐align software. CytX from SAR11 has the highest TM score with the *Arabidopsis thaliana* PLUTO protein, compared to other members for the LCOG1953 orthogroup.
**Table S4.** Included Sodium:Solute Symporters/ThiV homologues from the Eggnog 6 orthogroup LCOG0591. Accessions and organism names for the ThiV homologues, along with the reason for inclusion of each protein, are shown.
**Table S5.** Pairwise TM scores of selected Sodium:Solute Symporters determined by mTM‐align software. ThiV from SAR11 has the highest identity with the SSSP protein from *Emiliania huxleyi* and other eukaryotic proteins rather than the prokaryotic proline symporters.
**Table S6.** Putative transmembrane domains in selected Sodium:Solute Symporters relative to SiaT crystal structure. ThiV is predicted to have all 10 conserved transmembrane domains compared with the SiaT protein. See Figure 4B for more information.

**File S1.** Multiple sequence alignment of SAR11 CytX and other PLUTO homologues from Eggnog 6 orthogroup LCOG1953. SAR11 CytX does not share unique or specific substrate‐binding residues of PLUTO when compared with positions 121, 219 and 318 in HyuP/Mhp1 from *Microbacterium liquefaciens*.

**File S2.** Multiple sequence alignment of SAR11 ThiV and other HMP/Thiamin Sodium:Solute Symporters from Eggnog 6 orthogroup LCOG0591. SAR11 ThiV shares multiple unique predicted substrate‐binding residues with other HMP/Thiamin symporters, when compared with confirmed substrate‐binding residues of SiaT from *Proteus mirabilis*. See Figure 4C for more information.

## Data Availability

All data is provided as a part of this article.
